# Stress and Workload Assessment in Aviation—A Narrative Review

**DOI:** 10.3390/s23073556

**Published:** 2023-03-28

**Authors:** Giulia Masi, Gianluca Amprimo, Claudia Ferraris, Lorenzo Priano

**Affiliations:** 1 Department of Neurosciences, University of Turin, Via Cherasco 15, 10100 Torino, Italy; lorenzo.priano@unito.it; 2 Institute of Electronics, Information Engineering and Telecommunication, National Research Council, Corso Duca degli Abruzzi 24, 10129 Torino, Italy; gianluca.amprimo@ieiit.cnr.it (G.A.); claudia.ferraris@ieiit.cnr.it (C.F.); 3 Department of Control and Computer Engineering, Politecnico di Torino, Corso Duca degli Abruzzi 24, 10129 Torino, Italy; 4 Istituto Auxologico Italiano, IRCCS, Department of Neurology and Neurorehabilitation, S. Giuseppe Hospital, Oggebbio (Piancavallo), 28824 Verbania, Italy

**Keywords:** stress, mental workload, aviation, pilots, psychophysical assessment, HRV, EEG, NASA-TLX, cockpit, real flight

## Abstract

In aviation, any detail can have massive consequences. Among the potential sources of failure, human error is still the most troublesome to handle. Therefore, research concerning the management of mental workload, attention, and stress is of special interest in aviation. Recognizing conditions in which a pilot is over-challenged or cannot act lucidly could avoid serious outcomes. Furthermore, knowing in depth a pilot’s neurophysiological and cognitive–behavioral responses could allow for the optimization of equipment and procedures to minimize risk and increase safety. In addition, it could translate into a general enhancement of both the physical and mental well-being of pilots, producing a healthier and more ergonomic work environment. This review brings together literature on the study of stress and workload in the specific case of pilots of both civil and military aircraft. The most common approaches for studying these phenomena in the avionic context are explored in this review, with a focus on objective methodologies (e.g., the collection and analysis of neurophysiological signals). This review aims to identify the pros, cons, and applicability of the various approaches, to enable the design of an optimal protocol for a comprehensive study of these issues.

## 1. Introduction

Stress is a neuroendocrine, autonomic, behavioral, psychological, emotional, and cognitive phenomenon that occurs to promote effective coping strategies in response to a stimulus, called a *stressor*, perceived as challenging. Since this is a complex and synergistic response that varies from subject to subject and from situation to situation, finding an unambiguous definition is still an open challenge. Nevertheless, the current literature provides sufficient background to claim that each stressor provokes specific reactions, and that the peculiarities of the individual subject then determine a unique response in terms of manifestation, duration, and intensity [1,2]. To summarize, stress can be defined by three elements [3]:

1. a condition of heightened excitability or arousal;
2. an experience perceived as aversive;
3. an experience perceived as beyond one’s control or unpredictable.

From another perspective, stress aims to maintain homeostasis against a stressor, also promoting long-term adaptation [4]. Stressors can be most varied, both exogenous and endogenous, and can produce a *top-down* or *bottom-up* stress response. Various types of stressors are observed and described in the literature [5,6]:

- Physical: originating from the physical state, e.g., intense physical activity, sleep deprivation, fatigue, pain, or medical emergency.
- Environmental: originating from the external environment, e.g., noise, vibration, extreme climatic characteristics (such as high temperature or humidity), abnormal gas concentrations, high G-exposure.
- Emotional: originating from intense emotional states related to life events, e.g., exams, social roles, criticism, unfair treatment, relationship breakdown, job loss, or the death of someone close. Moreover, traumatic memories may provoke intrusive thoughts affecting the subject’s psychophysiological state.
- Mental/task-related: originating from the mental effort given by a task in terms of memory and attention allocation or task-related scenario (e.g., time available, confusion of instructions).
- Chronic: originating from a chronic life condition, e.g., severe financial difficulties, precarious/unstable employment, chronic illnesses or disabilities to the person or family members, or marital difficulties.

Recently, stress has been described by the World Health Organization as “the epidemic of the 21st century”, and its consequences on health have been widely documented. In the case of exposure to chronic stressors, such as those that may result from the subject’s occupation, most of the research is conducted via occupational health/medicine. In this respect, prolonged exposure to psychosocial stress is correlated with an increased likelihood of developing depressive disorders and cardiovascular or musculoskeletal diseases [7]. Stress is also correlated with increased demands for sick leave and decreased performance, due to its effects on cognitive abilities, directly impacting safety and costs [8]. In particular, impaired attention, slower reaction time, reduced vigilance and problem-solving, and impaired memory have been reported [8,9].

As already mentioned, the stress response consists of several elements. On a neurophysiological level, objective and quantitative measures can be obtained. However, it must be considered that stress is also an emotional experience, and consequently involves the enactment of specific and recognizable motor expressions and behaviors, so these aspects (e.g., facial expression, limb and neck movement, eye movement, dexterity) can also be measured with physical signals. A pivotal role is played by three different systems [10]. First, the hypothalamus–pituitary–adrenal (HPA) axis characterizes the so-called *slow* response. Second, the renin–angiotensin system (RAS) is known to be involved with a complex cascade [11]. Lastly, the autonomic nervous system (ANS), in particular the sympatho–adrenomedullary axis, is the most responsible for the *fast* response. The fast response allows the body to rapidly converge energies in a strong physical reaction. Therefore, noradrenaline is released, provoking an augmented heart rate, breathing rate, body temperature and sweating, auditory and visual exclusion phenomena, and vasodilation. Stress induced by physical activity follows strictly this response, while psychological stress is more complex, and regulatory mechanisms can produce opposite effects, such as vasoconstriction [12]. Moreover, it seems that exposure to mental stressors could be responsible for impaired neuromuscular performance [13]. Furthermore, perceptive, cognitive, and motor performance is affected by the stress response, through a more complex relationship. On the one hand, focusing both the body and the mind on a specific task may improve performance, excluding non-useful functions or less-relevant external stimuli. On the other hand, this might bring about huge side effects in complex environments, because of the lack of situational awareness (SA) and the impairment of decision-making. The relationship between stress and performance was traditionally described as an inverted U-function, i.e., performance increases with stress until a certain level of stress is reached, and from that level, performance declines. However, this is not acknowledged as an accurate model anymore, due to the complexity of factors to consider [14,15]. The effects of stressful task repetition, involving different stressors, such as in a work environment, must be disentangled, as well as the best training needed to cope with such stimulation. Hence, changes in physiological signals and performance that occur in these situations are only partially known [16,17].

In aviation, stress is a relevant topic because of its impact on human performance. Stress, mental workload, fatigue, distraction, and situational unawareness can be the cause of human errors, and produce a variety of scenarios, from small inefficiencies to great disasters [18]. Moreover, the long-term effects of flight stress exposure have been found to consist of post-traumatic stress disorder, anxiety disorder, depression, back pain, and neck pain [19,20]. Stress, workload, anxiety, and attention are linked by a complex relationship, which interfaces with a varied environment. Therefore, it is impossible to study stress as an isolated item, especially in the case of aircraft pilots. Even in civil pilots, stressors can derive from aircraft handling, especially during emergencies, environmental factors (e.g., temperature, noise, vibrations, G-exposure), shifts and sleep schedules, personal events, and interaction with other crew members. In a military environment, these stressors are exacerbated, and military personnel are selected and trained to face stressful situations and emergencies. However, many studies in the field have highlighted that there is still room for improvement [21,22], also considering the peculiarity of handling multiple tasks during flight missions. This requires the maintenance of good decision-making skills, SA, and physical readiness simultaneously, under a great variety of stressors. Moreover, a fine understanding of the pilot workload condition during different tasks and the continuous interaction with the onboard instrumentation available can be pivotal in the development of new aircraft technologies. In fact, next-generation cockpits are expected to feature virtual piloting and artificial intelligence [23]. For instance, single-pilot or unmanned aircraft have entered the market [24,25], fueling the interest in this research field. Nevertheless, it is challenging to move from a stress study in the laboratory to one in a real-life scenario, where several stressors may overlap, and to extract a reliable and robust omnicomprehensive model of this phenomenon.

From this perspective, technologies may increasingly support pilots in carrying out flight missions in complex scenarios. However, the early detection of alterations in a pilot’s psychophysical state, induced by stress and excessive mental workload, remains crucial to ensure pilot safety and avoid critical situations. To this end, various approaches can be considered in stress and workload assessment. Historically, these approaches are categorized as [26,27]:

- Self-assessment;
- Performance assessment;
- Objective assessment.

In this review, assessment techniques employed in the aviation field are collected that focus on pilot stress and workload. In Section 2, the review criteria are outlined. Section 3 reports the results of the literature search and presents an overview of stress and workload assessment approaches employed in the state of the art. Moreover, differences between studies conducted with real and simulated flight tasks are highlighted. The approaches employed in the selected studies are then categorized and described as self-assessment methods (Section 3.1), performance assessment methods (Section 3.2), and objective assessment methods (Section 3.3). Finally, special attention is paid to objective assessment, due to the broader spectrum of its applications, as further discussed and highlighted in Section 4 and Section 5.

## 2. Materials and Methods

A literature search was performed through the following online databases: Web of Science, PubMed, and Scopus. The search focused on published studies regarding the assessment of stress and mental workload in aircraft pilots, considering both civil and military fields. Therefore, as specific inclusion criteria, the target population was restricted to aircraft pilots with previous flight experience or cadets with advanced experience in simulator piloting. This choice derived from the intention of evaluating stressors and mental strains regarding flight tasks and associated stressful scenarios, not related to inexperience or discomfort. Moreover, pilot training provides stress management skills that could differentiate the physiological reactions of trained pilots from the general population. The employed search criteria were:

- Customized queries using keywords and Boolean operators in the form “Pilot (stress OR workload) in (aviation OR simulated flight OR real flight)”
- Year range restriction of 2012–2022.
- Writing language limitation to English.

After selection, the eligible articles were first categorized according to the type of flight task or scenario: simulated or real. Then, the analysis of the main stress and mental workload assessment methods in the articles was carried out. In the first place, objective assessment, self-assessment, and performance assessment were distinguished. Lastly, objective assessment approaches were further investigated, considering the instrumentation used as well. The strengths, weaknesses, and aims of the different approaches were also discussed. To easily categorize article information and display the data in figures, the Microsoft Office 365 package in 2021 edition (Microsoft, Redmond, WA, USA) and MIRO.com [28] (accessed on 23 December 2022) were exploited.

## 3. Results

A total of 48 articles were selected out of more than 400 results in the three online databases. After excluding the duplicates and after a preliminary title screening, abstracts were evaluated, producing a selection of approximately 90 studies. A full-text reading, and the imposed limitation about the subjects investigated, further reduced the set to the final 48 selected works.

Considering separately the studies that focus on a simulated flight task and those on a real flight task, an analysis of the methodological approaches was conducted. The studies that analyze the case of a simulated flight task will be referred to as simulation task articles (ST articles), while those that involve a real flight task will be referred to as real task articles (RF articles). For each article, the presence of objective, self, and performance assessment methods was then retrieved. Details about employed approaches and instrumentation are presented in the following subsections.

**Figure 1:**
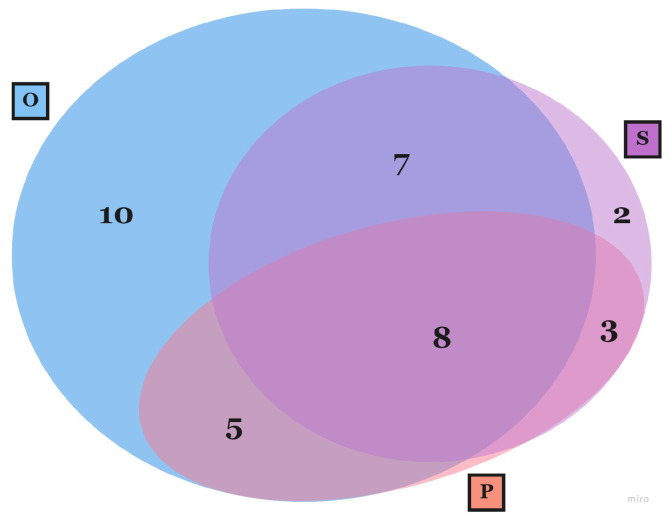
Venn Diagram representing the number of selected articles that focus on a flight simulation task (ST articles), using: Objective (O), Self (S), Performance (P) assessment approaches or their combinations.

35 ST articles were found [29,30,31,32,33,34,35,36,37,38,39,40,41,42,43,44,45,46,47,48,49,50,51,52,53,54,55,56,57,58,59,60,61,62,63]. In this context, the simulation of a flight task enables the control of experimental stimuli, such as the addition of engine failures (e.g., [43]), turbulence (e.g., [35]) or visibility setting (e.g., [30]). In addition, it allows for enhancement of the workload of the task by the addition of secondary subtasks, as in [57]. The combination of several methodological approaches for stress and workload assessment, as revealed by the analysis of ST articles, is shown throughout the Venn diagram representation in Figure 1. Looking at the diagram, it is evident that the use of multiple approaches simultaneously is common. Specifically, 23 out of 35 articles combined more than one type of evaluation method. Among them, eight combined all three types of approaches classified in this review (objective evaluation, self-assessment, and performance evaluation). Overall, objective evaluation is the most widely adopted approach, with 30 out of 35 studies applying it. Interestingly, performance appraisal is never used as a standalone method for stress and workload assessment.

**Figure 2:**
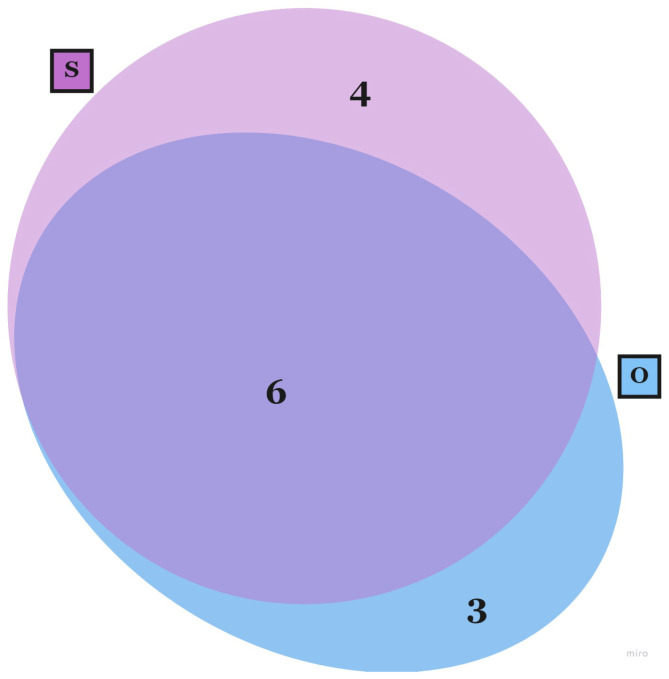
Venn Diagram representing the number of selected articles that realize a real flight task (RF Articles), using: Objective (O) or Self (S) Assessment in different combinations. No RF articles employing a performance assessment (P) approach were reviewed.

Regarding the RF articles, 13 were found belonging to this category [64,65,66,67,68,69,70,71,72,73,74,75,76] and the Venn diagram in Figure 2 shows the trend in the use of the different types of assessing methodologies. The multi-approach is again the most used. However, the performance analysis does not seem to be employed in real flight scenarios. On the contrary, the self-assessment approach is more frequently used also as a standalone method (4 out of 10 articles). This appears in contradiction with the previous result related to ST articles, where self-assessment is mostly employed with others measures. The severe difficulties that arise from a real scenario may be an explanation for this result. For instance, the safety of the pilots and the aircraft has to be ensured, as well as the confidentiality of flight data. Moreover, instrumentation and flight setup must comply with several regulations. Therefore, research protocols applied in real flight scenarios must ensure the significance and importance of the study before being conducted, provoking a bottleneck in published studies related to innovative technologies.

### 3.1. Self-Assessment

Self-Assessment (S) methodologies were employed in 20 ST articles [30,32,35,37,38,39,40,41,42,43,45,46,47,48,50,53,54,55,57,63] and in 10 RF articles [64,65,66,67,69,70,71,73,74,75]. Most of these studies used the Nasa–Task Load index (Nasa-TLX) [77], which is computed from a simple and strongly validated questionnaire, which is administered after task execution. The questionnaire inquires about the perceived workload in the domains of mental, physical, and temporal demand, performance, effort, and frustration. Due to its strong reliability and simplicity, it is used as a standalone method, as well as a ground truth to validate different systems for workload assessment [78]. The major limitation of Nasa-TLX is that it does not provide a real-time index, therefore it is not possible to immediately evaluate the effect of a single or short-term stimulus. Moreover, as in all the self-assessing techniques, the results can be influenced by the prior condition of the subject or other external stressors, such as social or peer pressure. For these reasons, self-assessment methods are often used in addition to objective or performance assessment tools, and they are combined with other tests for evaluating the general psychophysical condition, considering anxiety or depression symptoms, self-confidence, and personality traits.

Another equivalent index is the Subjective Workload Assessment Technique (SWAT) (e.g., in [45,75]), which evaluates workload in terms of time, mental, and psychological stress loads [79]. Lastly, another employed index is the Modified Cooper–Harper scale (MCH) [80], originally studied to evaluate the handling properties of an aircraft, which correlates the handling difficulties perceived with the mental workload experienced. For instance, this scale was employed in [35], and its modified version, the Bedford scale [27], was employed in [37]. Sometimes, several questionnaires and objective features are combined. For instance, in [38], the authors compared NASA-TLX, MCH, and some heartbeat features in assessing the mental workload of Air Force pilots during a simulation. Their results showed a strong correlation between Nasa-TLX and MCH and found all the measures computed able to differentiate the majority of the task conditions proposed in the simulation.

Other customized, non-structured questionnaires are also administered in some experiments, mainly inquiring about perceived anxiety, stress, strain, and fatigue during the execution of the specific task, as in [43,53,73]. These post-task questionnaires may also be useful in a multi-approach evaluation, to interpret the trends in the recorded physiological signals or to evaluate which factors are most critical for the subject during a task, hence performing a more thorough analysis of the task. For instance, the effects of the individual experience on physiological signals could be highlighted and distinguished from the task workload response. Otherwise, they can collect information about the population’s general condition (e.g., the effects of flying for a specific amount of hours a week, shifts, perceived satisfaction), as in [64,65].

Lastly, to overcome the previously mentioned limitations of self-assessment approaches, some real-time self-assessing techniques were introduced in the literature as well [81]. These studies commonly use additional devices, such as a keyboard, to self-score the perceived workload on-demand or through manual annotations by a test supervisor. The use of instantaneous self-assessment (ISA) was also found in this literature review, as in [63]. In this study, the pilots were flanked by the authors, who were in charge of reporting notes and observations, following the US Air Force School of Aerospace Medicine Crew Status Survey (CSS).

Moreover, looking again at Figure 2, four studies employed self-assessment methods alone in real flight conditions. Among them, two studies were meant to identify stressors and the criticality of normal duty in the case of helicopter emergency service pilots [64] and of short-haul and long-haul pilots [65], through cross-sectional broad-spectrum surveys. In the administered questionnaire, the authors were interested in investigating the satisfaction, the effects of shifts, the general well-being, symptoms of strain, and the main stressors and resources. However, in [66], the authors considered the emergency helicopter maneuver performed during the mandatory re-training of 10 volunteered pilots as the task. They assessed the workload due to the task, administering the Nasa-TLX questionnaire and comparing it between participants, also considering the annotations of pilot trainers regarding the performed maneuver and the reviewed emergency procedures. Lin et al. [67], instead, associated the Nasa-TLX with the assessment of SA. Specifically, they considered a helicopter rescue mission to a specific target, thus assessing SA in real time during the task, by the administration of a set of questions about the environment and, finally, analyzing answer correctness and the response time. Finally, they highlighted the relationship between SA maintenance in high workload conditions and the expertise of the pilots.

### 3.2. Performance Assessment

Performance assessment involves a double perspective: the evaluation of the “goodness” of the executed task and the modeling of the expected performance, considering different input data (e.g., heart rate, workload level, task features). The analysis of the selected papers revealed that 16 out of 48 articles made use of this kind of analysis [30,31,32,35,36,37,39,42,43,46,48,49,50,51,54,56], all belonging to ST articles. The goodness of an execution of a task can be assessed via the evaluation of the strictness in following the instructions. For instance, maintaining a certain altitude is an objectively measurable quantity that can define a such property. In [32], Maneuver Error Index (MEI) is proposed in this sense. It evaluates the heading (deg) and the true altitude (ft) in different flight segments. These kinds of measures can be useful also in the evaluation of risk-taking during flight task execution, as done by Wang et al. [49]. The authors divided the subjects into groups (high-risk, low-risk), based on the lowest altitude reached in a specific task segment. Furthermore, the behavior in front of a challenging task, in terms of risk-taking, may be also strongly influenced by SA. Therefore, considerations about SA should be taken into account in this picture. Still, SA is usually assessed with a set of questions about the current flight situation administered during the flight execution—e.g., questions about altitude, fuel, and best maneuver features to be performed [82]. Therefore, this approach is difficult to superimpose on other evaluations because it inherently involves the addition of a supplementary workload.

The frequency and the type of particular maneuver executed can be used as a performance metric, too. In the case of flight simulators, some of these metrics can be easily saved, such as accurate operation rate and reaction time [39]. Moreover, further subtasks can be introduced and scored. In real flights, instead, where these kinds of metrics cannot be easily evaluated and stored, an alternative approach involves external assessment by an expert pilot or instructor, which gives an evaluation of the flight performance [83]. External evaluation from an expert is sometimes used in simulation as well, as in [36].

The influence of workload on performance became relevant with the introduction of automation technology, especially in the military field [84]. New technologies are needed to ensure a simplification in procedures and the maintenance of safety. Therefore, a precise evaluation of the single task or instrumentation handling effect was necessary. In other words, it became necessary to know the type and level of workload that causes performance impairment. Therefore, complex models of workload estimation have appeared in the literature. A milestone in the military field was set by Aldrich et al. [85], who proposed the VACP model (visual, auditory, cognitive, psychomotor), based on the division of the task demand in different kinds of resources/channels. Each task can be scored according to the use of all the channels (visual perception, auditory perception, verbal cognition, spatial cognition, manual response, and speech response) to obtain a workload index [86]. This model was taken as a gold standard in the following years. For instance, the authors in [46] employed different subjective scales and the VACP model to assess the workload of 22 commercial-aviation pilots. In particular, in this study, the VACP model was integrated with a questionnaire in the so-called Behavior–Cognitive Model Scale.

### 3.3. Objective Assessment

Objective assessment of stress and workload relies on the observation of the psychophysical reaction provoked by stressors. As already mentioned, autonomic and hormonal regulations play a key role in this response and can be observed in terms of many physiological signals. There are also behavioral and secondary effects that can be observed and measured, furnishing other possible objective measures. An extensive description of the main computed features regarding objective measures in this context is available in the literature [5,87,88,89,90,91,92,93,94]. In addition, in ref. [95], there is an interesting summary of the expected behavior of cardiorespiratory, cerebral, and eye activities in the presence of workload, attention, and fatigue.

**Figure 3:**
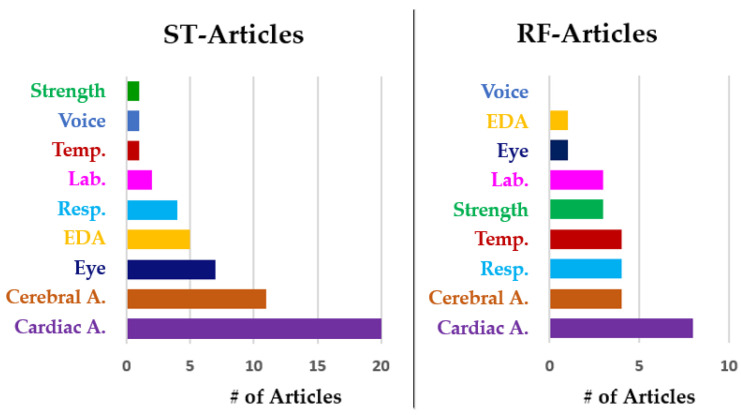
Objective measures employed in ST articles (with simulated flight task) and RF articles (real flight task).

Objective assessment was employed in 30 ST articles [29,30,31,32,33,34,36,38,39,40,41,42,43,44,45,47,48,49,50,51,52,54,55,56,57,58,59,60,61,62] and in 9 RF articles [68,69,70,71,72,73,74,75,76]. As a result of the analysis of these studies, the performed objective measures, the calculated parameters, and the employed instrumentation are summarized in Table 1. Moreover, Figure 3 is provided to highlight the trend in the employment of different objective measures across articles. The distinction between RF articles and ST articles allows for a better assessment of the differences determined by the two conditions.

As shown in Table 1 and Figure 3, cardiac activity (Cardiac A.) measures were the most employed across all studies by far and, in 14 out of 48 articles, the Cardiac A. observation was the unique objective measure performed. In further detail, the electrocardiogram (ECG) is the most complete and reliable instrument for recording cardiac activity (Cardiac A.), because it allows for the extraction of several additional pieces of information other than the heartbeat, which retains a relevant clinical significance. ECG records the electrical activity of the heart muscles, composed of a cycle of atrial and ventricular polarization and depolarization. The signal has known morphological and temporal characteristics that allow for a very deep understanding of heart functionality, e.g., the main wave in ECG is clinically called the QRS complex. In the context of stress and workload estimation, the main features considered are the heart rate (HR) and the heart-rate variability (HRV) instead of the QRS complex characteristics. The sympathetic and parasympathetic nervous systems increase and suppress heart rate, respectively, determining HRV. Indeed, it is the most studied feature for stress evaluation, considering its time domain, frequency domain, and nonlinear features [96]. Other than HRV, another cardiac activity-related measure is blood pressure, which is employed by the authors in ref. [76]. Regarding technological aspects, single-lead ECG, chest-strap heart-rate monitors, and photoplethysmogram are the most commonly employed instruments to monitor HR and HRV in aviation. The constraints of the specific recording environment (i.e., the cockpit or simulator) may also play a role in the choice of devices.

Since ANS acts at the cardiorespiratory level, breathing rate, lung capacity, and blood saturation are also metrics of interest. These measures are grouped in the category Resp. in the subsequent sections and in Table 1. A spirometer, pulse oximeter, and resistive sensors were employed for recording these parameters.

Electrodermal activity (EDA), sometimes called galvanic skin response, skin conductance, or resistance, is another measure influenced by the ANS dynamics. Sweat gland activity is reflected in the electrical properties of the skin and it is regulated by the sympathetic nervous system, not only to maintain thermoregulation but also in many other mechanisms, including emotional arousal and stress response [5,97]. EDA is commonly used in the definition of valence and arousal for emotional state evaluations. The canonical approach to EDA analysis consists of studying the signal in terms of tonic and phasic activities: tonic expresses the slow changes in the signal and gives a mean level of conductance, and phasic is composed of transient responses. The latter is investigated as a single-stimulus response or non-specific response in multi-stimulus contexts, considering the rate of responses in certain time intervals [98]. For instance, the authors in [31] computed the mean tonic and phasic activities, providing significant differences in mean tonic activities between different simulated flight maneuvers. The authors in [44] used EDA to distinguish sympathetic from parasympathetic ANS activation, using EDA combined with cardiorespiratory activity recordings. The latter is influenced by sympathetic and parasympathetic activation combined. However, the authors do not provide results regarding the EDA activity. The authors in [54] correlated the behavior of EDA activity in two simulated fight tasks, where one was sided with an additional social stressor. They found differences in the signal behavior, depending on some personality traits of the pilots.

Skin temperature (Temp) is also considered a traditional measure for stress assessment [99,100], and is also employed in the aviation domain. Thermistors, thermal cameras, and infrared thermometers may be used in this context as instrumentation. The authors in [59] measured mean skin temperature from the subject’s arm via a skin temperature sensor. The authors also considered EDA and cardiac, respiratory, and muscular activities. However, the mean temperature was excluded from the subsequent optimized multi-modal features analysis, because it was found to be less relevant. The authors in [70,71,73] measured temperature using infrared thermometers before and after the execution of a flight task. The authors in [76] obtained finger temperature measurements together with blood pressure. The authors defined a measure of arousal called the psychophysiological arousal value (PAV), obtained from typical autonomic response patterns clustering and eigenvectors of the physiological data obtained by exploratory factor analysis [101]. They collected data from pilots performing different simulated tasks. Computing PAV, they were able to identify the most challenging tasks (i.e., air-to-air refueling) and differences in the reactions between novices and professionals. In addition, they found reactions in simulated flights most often compared to those in real flights, unlike the results in [69,75].

Autonomic function affects pupil behavior, and, therefore, pupillometry, is part of eye-activity evaluation, together with the analysis of scanning patterns and blinking during the execution of a task. These, indeed, are deeply influenced by the behavioral effects of concentration, anxiety, workload, and stress. People tend to fixate on objects of interest appearing in the visual field via saccades (overt attention). Thus, in this context, it is important to know whether object-based selection occurs for overt attention and its response time. Therefore, eye-tracking systems, able to provide information about fixation, blinking, space scanning, and saccades, are also frequently found in this type of investigation. The analysis revealed that eight out of 48 articles provide measurements of eye activity. For instance, the authors in [29] used eye activity to continuously assess anxiety during a flight simulation, to better understand the relationships between performance and level of training, and the authors in [47] assessed the inside/outside fixation rate to appreciate the visual scanning technique in relation to workload and experience. In addition, the authors in [58] proposed a simulation of an unexpected in-flight event and were able to identify a strong reduction of the scanned space in the presence of a such stressor. Finally, the authors in [31] measured fixation and saccade behavior as fixation frequency, mean fixation time, saccade frequency, mean saccade time, and mean pupil diameter was found to be pertinent to workload level.

Cerebral activity is peculiar in stress reactions. Activity in various cerebral areas is intensively elicited in a flight scenario, and Event-Related Potentials (ERP) can be detected using electroencephalography (EEG) or near-infrared spectroscopy (NIRS) signals with different configurations and numbers of channels. For instance, the authors in [62] employed functional NIRS to evaluate prefrontal cortex activation throughout signal feature extraction. Looking at Figure 3, Cerebral A. seems to be commonly employed for this purpose. Deepening the analysis of the articles, it turns out that three out of four RF articles implemented cerebral activity evaluation using the same experimental protocol. The protocol includes a pre-flight and a post-flight evaluation (pre–post) and the main focus is on cortical arousal and fatigue, assessed by the critical flicker fusion threshold [102], as in [70,71,73]. On the other hand, the use of EEG and NIRS appears in some ST articles. This is probably due to the difficulty of recording good-quality cerebral activity in a cockpit during real flight time. Movement artifacts and the possible use of safety helmets may augment the already great complexity of recording cerebral activity. Partially encouraging results in this regard were obtained by Dehais [68], where an EEG helmet with six dry electrodes was used during the real flight of the ISAE-SUPAERO (Institut Supérieur de l’Aéronautique et del’Espace-French Aeronautical University in Toulouse, France) experimental light aircraft. They were able to provide oddball audio stimuli to the pilots through the pilot’s aviation headset (Clarity AloftPro), while the pilot was monitoring the flight (low load condition) and when the pilot was piloting the aircraft controlled by the flight instructor. They found statistically different results by looking at ERPs and spectral power analysis in the two presented situations. However, the employed automatic classification showed the necessity of further hardware refinement because of the difficulties in achieving high accuracy.

Voice frequencies and speech can be affected by stress [103,104], but this review found only one study introducing voice analysis. The authors in [52] provided a database correlating heart rate and the voices of eight airline pilots during cognitive solicitation in a full flight simulator. The voice recordings were done via the headset’s built-in microphones and then transmitted in glass fiber after proper processing.

Furthermore, the complex stress response has a direct influence on muscle strength and movement. For instance, the *fight-or-flight* response allows the body to physically hyper-perform, while fear or concentration can lead to general immobility of the body. Electromyograms (EMG) can provide information about muscle condition and strength. For instance, one was employed in [59]. Other kinds of measures consider exercise set performance, such as jumping sets, or isometric hand strength measures to confront the strength condition and physical prowess before and after a task. An example of this kind of evaluation is in the protocols of [70,71,73], in the RF articles group, where a wide range of objective evaluations was used in a pre–post analysis fashion. They involved the assessment of: Cerebral A. in terms of cortical arousal; limb strength in terms of horizontal jumps sets and isometric hand grip; Temp. as skin temperature; Resp. in terms of blood oxygen saturation and spirometry measures; laboratory analysis (Lab.) of blood lactate, urine color chart, urine protein, glucose, nitrates, pH and specific gravity. Laboratory analysis of body fluids such as blood, saliva, and urine can provide information about the occurrence of stress response and ANS activation. For instance, cortisol is a well-known stress hormone and can be measured in both blood and saliva, even though its concentration may vary during the day independently from stress [105]. Blood samples can also furnish white blood cell count, endocannabinoids, and lactate concentrations. Urine samples have been examined for hydration level (color), proteins, glucose, noradrenaline, pH, and specific gravity. All these chemical compounds and physical measures can directly show if a neurophysiological stress reaction has happened, considering the metabolic outcomes, but in a post-analysis fashion only [106]. Lastly, Cardiac A. was measured as heart rate by a pulse-oximeter system [73] and as heart rate and heart-rate variability [70,71] using a Polar V800 smartwatch (POLAR, Kempele, Finland). In particular, in these last two articles, the HR measurements were maintained during the flight execution and were not limited to a pre–post analysis. Moreover, all three studies considered military personnel. In particular, the authors in [71,73] focused on helicopter crews and compared their responses to the control group (civil subjects) when performing different maneuvers: a rescue crane maneuver and a low-altitude flight maneuver for the first study; two night flights and two instrument flights in the second. The pre–post analysis was able to identify the effects of the different flight tasks and the experience of the personnel in terms of psychophysical response.

## 4. Discussion

Most of the selected articles relied on a multi-approach stress assessment based on different methods, as previously shown in Figure 1 and Figure 2. Many approaches revealed their potential for stress and workload monitoring on pilots.

Surveys and self-assessment approaches are preferred to identify the general pilot conditions concerning the work environment (e.g., shifts, satisfaction) or the effect of a specific task (e.g., landing is perceived as more stressful than heading). Nasa-TLX is the most used self-assessment method due to its reliability and simplicity. Therefore, it is often used as a ground truth for the workload level.

Performance assessment is used as a complementary tool to self and objective assessment. This can be explained by the fact that performance impairment is a crucial aspect of workload and stress response, therefore performance assessment enables the pivotal design of predictive models that relate workload/stress to the outcoming performance. By taking combined measures, it is possible to correlate the behavior of objective or self-assessment measures with variations in performance. However, performance assessment as a standalone method has no predictive power on the internal psychophysiological conditions of the pilot.

Focusing on objective assessment, different measurements of body activity were taken into account simultaneously in most of the studies. It was already mentioned that objective assessment uses several instruments and hinges on the observation of different objectively quantifiable phenomena. Cardiac A. evaluation is one of the most descriptive of stress conditions. It is also a measure easily transferable in the cockpit environment, due to the availability of lightweight and convenient recording devices. There is no doubt about the power of Cardiac A. as a descriptor of stress, workload, and emotional state; however, the significance of the results obtained in a controlled environment, such as a simulator, is not automatically equally powerful in a real flight condition, as shown from the results in [69,75]. Nevertheless, experimental controllability is higher in simulated flight tasks and a broader spectrum of monitoring tools is evaluated in this case, with a preference for real-time monitoring evaluation.

Looking at Figure 3, Cerebral A. is also commonly employed in the selected articles. However, in a real flight scenario, only the authors in [68] measured cerebral activity in real time, while others performed a pre–post evaluation. Due to the potentiality of the cerebral activity analysis, which is reflected in the use of EEG and NIRS during simulated tasks, it seems reasonable to claim that recording issues discourage the use of cerebral activity assessment in real flight.

Eye activity was unexpectedly not employed in RF articles, whereas it is widely investigated in this field due to the possibility of identifying scanning and fixation patterns, which may be relevant to assessing the use of instrumentation, target/danger identification, and verifying the optimal setup in the cockpit. In more detail, eye-activity assessment typically aims at pilot interface optimization (e.g., optimized instruments position or virtual command setup) or the study of the sky-scanning techniques for some critical maneuvers. Studies summarizing knowledge in these techniques can be found in the literature, such as the use of eye-tracking to infer cognitive state and increase safety. In ST articles, good results were provided by the use of eye-tracking. In contrast, in real flight conditions, there seem to be some results in the literature in terms of using eye-activity assessment for flight-phase recognition [107,108], but not for the purposes investigated in this review, to the authors’ knowledge. A similar trend is followed by voice analysis, which gave promising results for stress detection [109] in aviation [52,110].

Moreover, it is very often necessary to have real-time measures of stress and workload; therefore activity that changes rapidly with the stress/workload condition, and which can be easily recorded and interpreted, should be preferred. In this direction, it has to be considered that laboratory sample analysis (Lab.) of saliva, blood, or urine can be only used in a pre–post task evaluation, as well as some type of limb strength evaluation. This could explain why, in ST articles, where real-time assessment is possible, these measures are infrequent. In real flight tasks, instead, an analysis of the situation pre-flight and post-flight or post-flight alone is preferred.

The main strength of the objective assessment approach is the possibility of performing real-time objective evaluation, which is easy to automate. The main limitation of these approaches derive from three aspects: the first one is an intrinsic limitation of the stress/workload detection problem and regards the complexity of the neurophysiological response, which can cause low specificity in event recognition (e.g., exceeding stress level can be confused with physical strain). The second limitation regards real-time approaches, which have to deal with a double-time resolution problem. Indeed, the speed of body response can be slower or faster depending on the stimulus (seconds to minutes) so the designed monitoring system should consider different time scales of analysis and carefully select proper features that may be computed in short and long epochs. The last aspect regards the constraints of aviation itself. Movement artifacts and the possible use of safety helmets may augment the already great complexity of physiological signal recording. In addition, for safety reasons, any modification of pilot equipment is subject to precise and severe regulation, and it is, therefore, usually a very long and difficult process to integrate new instruments into certified equipment for research purposes. Technical requirements, intrusiveness, and operator acceptance are severe constraints in this scenario.

**Figure 4:**
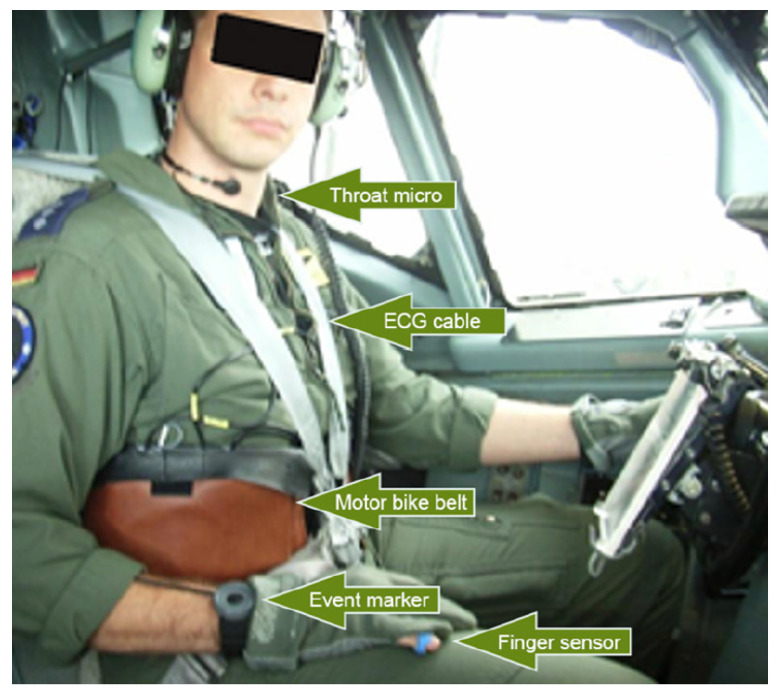
Instrumentation setup employed and shown in [76] for real-time monitoring. The authors registered a single-lead ECG, finger temperature, pulse wave, and EDA.

To overcome the mentioned limitations and as a result of the analysis of the selected articles, it can be claimed that the instrumentation used for real-time monitoring in RF articles should be light and comfortable, e.g., a single-lead wearable ECG or a smartwatch. Indeed, Cardiac A. is monitored in 12 out of 13 real-time approaches, since it may be measured without cumbersome instrumentation. Resp. and eye-activity measurements are also recorded in real time in [74], but the cockpit setup is not shown in this article. The authors in [76] made use of Cardiac A., EDA, and temperature activity monitoring. The setup used in the cockpit for real-time monitoring is shown in Figure 4. The aircraft is a heavy-class E-3 Sentry, which is a modified Boeing 707 aircraft. As already mentioned, they computed PAV, from the analysis of the signals. Then, they inferred the psychophysiological status of the pilots in relation to the performed task (e.g., real and simulated normal flight, approach, landing) and the experience of the pilots.

Furthermore, methodologies enabling the real-time assessment of limb strength are appearing in the literature. For instance, the grip force measure has been studied in relation to stress [111], and Wagner et al. [112] measured the grip force on a control stick during a tracking task. They were able to appreciate an increment in the grip force corresponding to increased task difficulty. Therefore, grip force could be considered to be a real-time objective measure to use during real flight, as long as the sensor is properly integrated with control commands. For instance, it could be of interest to evaluate the grip force on the cloque, as well as head movements to monitor postural behavior. The use of dynamometers and inertial sensors may be considered in this direction. However, its significance in a real flight condition evaluation is yet to be verified.

## 5. Conclusions

In this review, studies exploring stress and workload in aviation were collected. In particular, the selected articles focused on monitoring a pilot’s condition in terms of psychophysiological state during simulated and real flight tasks. This topic has great relevance for multiple reasons. First, safety can be improved by monitoring the lucidity of the pilot; second, the pilot’s well-being and work environment can be ameliorated, by understanding and limiting stress exposure. Lastly, finely measuring the effects of different tasks/subtasks may enable the optimization of instrumentation, in the direction of virtual/automatic solutions. The analysis of the 48 selected articles highlighted the main approaches to stress and workload assessment in various experimental conditions. Objective assessment—i.e., the collection of biological quantitative measures—is the most employed. In particular, cardiac activity is the most interesting, usually assessed in terms of HRV.

The main issues that emerge from this review are linked to the mutual interaction of different aspects, stressors, and stimuli during a complex task, such as a flight task. Recording limitations arise from the integration of new instrumentation in the cockpit and the level of quality in signal recordings that can be achieved. Studies providing real-time measurements of psychophysiological conditions prefer light and non-cumbersome instrumentation, which is easily wearable by the pilot above or underneath their suit, as shown in Figure 4. Finally, it must also be mentioned that confidentiality is crucial in this field.

To conclude, real-time stress and workload monitoring, with application in stress and workload prediction, seems to be viable only through objective assessment approaches, such as the recording of electrophysiological signals. These measures have demonstrated their power in this direction, but few results are applied in the aviation context, probably due to previously mentioned issues. Up until now, the majority of results conduce pre-flight and post-flight pilot monitoring in real flight conditions. The few real-time measures performed cardiac activity monitoring, computing HRV. Nevertheless, several alternative research directions (e.g., eye-tracking, muscular activity evaluation through ECG) appear promising and could provide further insight into the detection of aviation pilot stress, once issues related to setup and safety regulations are solved.

## Figures and Tables

**Table 1:** Objective Measures performed in the reviewed articles, type of analysis, and instrumentation employed.

Measures	Parameters	Intrumentation	Refs.
Cardiac A.	HR ^1^; HRV (temporal, frequency and nonlinear features); Frequency bands; blood pressure.	Single-lead ECG; Chest strap; PPG.	ST articles: [30,31,32,33,34,36,38,39,44,45,48,49,52,55,56,57,58,59,60,61]; RF articles: [69,70,71,72,73,74,75,76]
Resp.	Breathing rate, lung capacity, and blood saturation.	Spirometer; pulse oximeter; resistive sensors.	ST articles: [31,44,59,61]; RF articles: [70,71,73,74]
Cerebral A.	Frequency bands, ERPs, reaction time	EEG; NIRS.	ST articles: [31,41,42,43,45,48,50,51,60,61,62]; RF articles: [68,70,71,73].
EDA	Mean tonic activity; mean phasic activity; rate of responses in time.	Wireless skin conductance sensor; Electrodes and amplifiers acquisition system for bioelectrical signals.	ST articles: [31,44,54,59,61]; RF articles: [76].
Temp.	Temporal and frequency features.	Infrared thermometers, skin temperature sensors.	ST articles: [59]; RF articles: [70,71,73,76].
Eye	Pupillometry (pupil diameter); mean blinking rate and duration; mean fixation rate and time; mean saccade rate and time.	Head-mounted tracker.	ST articles: [29,31,43,47,48,58,61]; RF articles: [74].
Lab.	Blood: white blood cells count, endocannabinoids and lactate concentrations; Saliva: Cortisol; Urine: hydration level (color), proteins, glucose, noradrenaline, pH, and specific gravity.	Multiple laboratory techniques.	ST articles: [40]; RF articles: [70,71,73].
Strength	Temporal and frequency features.	EMG.	ST articles: [59]; RF articles: [70,71,73].
Voice	Frequency bands; Pitch frequency.	Microphone.	ST articles: [52].

^1^ HR: Heart Rate; HRV: Heart Rate Variability; ECG: Electrocardiography; PPG: Photopletophismography; EMG: Electromyography; EEG: Electroencephalography; ERP: Event-Related Potential; NIRS: Near-Infrared Spectroscopy; ST articles: articles presenting with a simulated flight task; RF articles: articles presenting with a real flight task.

## Data Availability

Not applicable.
