# Correction: Masi et al. Stress and Workload Assessment in Aviation—A Narrative Review. *Sensors* 2023, *23*, 3556

**DOI:** 10.3390/s24020690

**Published:** 2024-01-22

**Authors:** Giulia Masi, Gianluca Amprimo, Claudia Ferraris, Lorenzo Priano

**Affiliations:** 1 Department of Neurosciences, University of Turin, Via Cherasco 15, 10100 Torino, Italy; lorenzo.priano@unito.it; 2 Institute of Electronics, Information Engineering and Telecommunication, National Research Council, Corso Duca degli Abruzzi 24, 10129 Torino, Italy; gianluca.amprimo@ieiit.cnr.it (G.A.); claudia.ferraris@ieiit.cnr.it (C.F.); 3 Department of Control and Computer Engineering, Politecnico di Torino, Corso Duca degli Abruzzi 24, 10129 Torino, Italy; 4 Istituto Auxologico Italiano, IRCCS, Department of Neurology and Neurorehabilitation, S. Giuseppe Hospital, Oggebbio (Piancavallo), 28824 Verbania, Italy

## Additional Affiliation

The published publication [1] had an error regarding Lorenzo Priano’s affiliation. In addition to affiliation 1, the updated affiliation should also include affiliation 4: Istituto Auxologico Italiano, IRCCS, Department of Neurology and Neurorehabilitation, S. Giuseppe Hospital, Oggebbio (Piancavallo), 28824 Verbania, Italy. The authors state that the scientific conclusions are unaffected. This correction was approved by the Academic Editor. The original publication has also been updated.
